# Sirolimus treatment for paediatric head and neck lymphatic malformations: a systematic review

**DOI:** 10.1007/s00405-023-07991-1

**Published:** 2023-04-28

**Authors:** Alberto Maria Saibene, Cecilia Rosso, Giovanni Felisati, Lorenzo Pignataro, Antonio Schindler, Giorgio Ghilardi, Giacomo Colletti, Michele Gaffuri, Francesco Mozzanica

**Affiliations:** 1 Otolaryngology Unit, ASST Santi Paolo e Carlo, Department of Health Sciences, Università Degli Studi di Milano, Via Antonio di Rudinì, 8, 20142 Milan, Italy; 2 Department of Otolaryngology and Head and Neck Surgery, Fondazione IRCCS Ca’ Granda Ospedale Maggiore Policlinico, Milan, Italy; 3 Department of Clinical Sciences and Community Health, Università Degli Studi di Milano, Milan, Italy; 4 Department of Biomedical and Clinical Sciences, Università Degli Studi di Milano, Milan, Italy; 5 Clinica Chirurgica Generale, Department of Health Sciences, Università Degli Studi di Milano, Milan, Italy; 6 Cranio Maxillo Facial Surgery, Università Degli Studi di Modena e Reggio Emilia, Modena, Italy; 7 Department of Clinical Sciences and Community Health, San Giuseppe Hospital, Università Degli Studi di Milano, Milan, Italy

**Keywords:** Vascular anomaly, Lymphangiomatosis, Paediatric mass, Rapamycin, Lymphatic malformation

## Abstract

**Purpose:**

This PRISMA-compliant systematic review aimed to assess risks and benefits of sirolimus treatment for paediatric lymphatic malformations by focusing not only on treatment efficacy but also on possible treatment-related adverse events, and treatment combinations with other techniques.

**Methods:**

Search criteria were applied to MEDLINE, Embase, Web of Science, Scopus, Cochrane Library, and ClinicalTrials.gov databases and included all studies published up to March 2022 reporting paediatric lymphatic malformations treated with sirolimus. We selected all original studies that included treatment outcomes. After the removal of duplicates, selection of abstracts and full-text articles, and quality assessment, we reviewed eligible articles for patient demographics, lymphatic malformation type, size or stage, site, clinical response rates, sirolimus administration route and dose, related adverse events, follow-up time, and concurrent treatments.

**Results:**

Among 153 unique citations, 19 studies were considered eligible, with reported treatment data for 97 paediatric patients. Most studies (*n* = 9) were case reports. Clinical response was described for 89 patients, in whom 94 mild-to-moderate adverse events were reported. The most frequently administered treatment regimen was oral sirolimus 0.8 mg/m^2^ twice a day, with the aim of achieving a blood concentration of 10–15 ng/mL.

**Conclusion:**

Despite promising results for sirolimus treatment in lymphatic malformation, the efficacy and safety profile of remains unclear due to the lack of high-quality studies. Systematic reporting of known side effects, especially in younger children, should assist clinicians in minimising treatment-associated risks. At the same time, we advocate for prospective multicentre studies with minimum reporting standards to facilitate improved candidate selection.

## Introduction

The International Society for the Study of Vascular Anomalies (ISSVA) classifies lymphatic malformations (LM) as low-flow vascular anomalies of the lymphatic system, often referred to with a misnomer such as lymphangiomas of cystic hygromas [1]. The incidence of LM is estimated to be 1.2–2.8 per 1000 births [2]. Around 50–60% of LM cases are present at birth, while approximately 80–90% will become evident by two years of age.

The pathogenetic mechanisms of LM are currently under debate [3]. However, recent findings of somatic genetic mutations in PIK3CA point to a developmental impairment of lymphatic channels due to improperly functioning endothelial cells [4]. The size of an LM usually increases proportionally in relation to the patient’s body growth. However, abrupt spurs are frequently observed, particularly following infection in drained tissues, trauma, and hormonal changes. Intracystic haemorrhages caused by lacerations of the septae where vessels run are another frequent cause of volume accretion. Frequently, all these occurrences are accompanied by acute symptoms such as pain, tenderness, and other signs of inflammation.

The most recent classification subdivides simple LM into macrocystic, microcystic, and combined LM [5]. Typical locations are the head and neck (approximately 60% of cases), proximal extremities, and trunk [6].

LMs may appear as part of a syndrome, including generalised lymphatic anomaly, central conducting lymphatic anomaly, Gorham-Stout syndrome, kaposiform lymphangiomatosis, and other diseases associated with PIK3CA mutation, such as PIK3CA-related overgrowth syndrome (PROS).

Clinical presentation depends on the site and size of the malformation. Involvement of the respiratory tract is of special concern, since such LMs can cause tongue extrusion, jaw deformity, swallowing issues, and speech difficulties, and even lead to life-threatening conditions that obstruct the upper airways [5].

Treatment options for lymphatic malformations are heterogeneous and include observation, sclerotherapy, radiofrequency ablation, laser treatment, and surgical excision. The overall aim of treatment is functional and cosmetic and is focused on reducing the psychosocial burden of patients and families [7, 8].

A personalised therapeutic approach is typically provided and depends on LM size, position, growth rate, and type, as spontaneous regressions have not been verified in the literature [9, 10]. Surgeries and sclerotherapy are usually effective for macrocystic LM, though size and position may reduce their feasibility and effectiveness. Conversely, surgery for microcystic LMs remains challenging due to their infiltrative nature [11]. Interstitial sclerotherapy has demonstrated encouraging microcystic LM results, with an approximate 50% reduction in volume [12, 13].

If more common options, such as surgery and sclerotherapy for LM, result in only modest efficacy or cannot be employed, rapamycin, also known as sirolimus, represents the current standard care for medical treatment.

Rapamycin is formally classed as a macrolide antibiotic drug. It owes its name to Rapa Nui (Easter Island) where it was first discovered from a soil sample containing *Streptomyces hygroscopicus*. The initial observed effect of rapamycin was modest antifungal activity. The drug was later adopted as part of a combination regimen for reducing the rejection of kidney transplants due to its immunosuppressant effects. It was subsequently studied as a potential drug for cancer since it demonstrated (weak) antiangiogenic effects. Sirolimus is active against the mammalian target of rapamycin (mTOR), a serine/threonine protein kinase considered a potential pathway of vascular malformation pathogenesis. The specific role of sirolimus in treating vascular malformations is linked to its ability to block the PIK/AKT/mTOR pathway [14].

The first case of LM treated with sirolimus was reported in 2011 [15]. Since then, many reports and case series have demonstrated the efficacy of sirolimus in reducing the size of LMs [16]. However, objective data on the effectiveness of this therapy are not currently available, particularly with regard to the paediatric population.

The present systematic review aimed to analyse current knowledge on the use of sirolimus as a treatment for LM in paediatric patients, not only in terms of its efficacy but also in terms of drug regimens and adverse events.

## Methods

### Search strategy

After registering with the PROSPERO database (ID CRD42022314066), we conducted a systematic review between March 3, 2022, and February 10, 2023, according to PRISMA reporting guidelines [17]. We carried out systematic electronic searches for studies in English, Italian, German, French, and Spanish that reported original data on sirolimus treatment for paediatric head and neck lymphatic malformations.

On March 3, 2022, we searched the MEDLINE, Embase, Web of Science, Cochrane Library, Scopus, and ClinicalTrials.gov databases for sirolimus and rapamycin in association with lymphovascular, cervicofacial, and paediatric search terms. Complete search strategies and the number of items retrieved from each database are provided in Table 1.

**Table 1:** Search strategy details and items retrieved from each consulted database

Database	Search date	Query	Items retrieved (*n*)
Medline	March, the 3rd, 2022	("sirolimus"[MeSH Terms] OR "sirolimus"[All Fields] OR ("rapamycin s"[All Fields] OR "rapamycine"[All Fields] OR "rapamycins"[All Fields] OR "sirolimus"[MeSH Terms] OR "sirolimus"[All Fields] OR "rapamycin"[All Fields])) AND ("lymphatic vessels"[MeSH Terms] OR ("lymphatic"[All Fields] AND "vessels"[All Fields]) OR "lymphatic vessels"[All Fields] OR "lymphatic"[All Fields] OR "lymphatic system"[MeSH Terms] OR ("lymphatic"[All Fields] AND "system"[All Fields]) OR "lymphatic system"[All Fields] OR "lymphatics"[All Fields] OR ("blood vessels"[MeSH Terms] OR ("blood"[All Fields] AND "vessels"[All Fields]) OR "blood vessels"[All Fields] OR "vascular"[All Fields] OR "neovascularization, pathologic"[MeSH Terms] OR ("neovascularization"[All Fields] AND "pathologic"[All Fields]) OR "pathologic neovascularization"[All Fields] OR "vascularisation"[All Fields] OR "vascularization"[All Fields] OR "vascularisations"[All Fields] OR "vascularise"[All Fields] OR "vascularised"[All Fields] OR "vascularities"[All Fields] OR "vascularitis"[All Fields] OR "vascularity"[All Fields] OR "vascularizations"[All Fields] OR "vascularize"[All Fields] OR "vascularized"[All Fields] OR "vascularizes"[All Fields] OR "vascularizing"[All Fields] OR "vasculars"[All Fields]) OR ("lymphangioma"[MeSH Terms] OR "lymphangioma"[All Fields] OR "lymphangiomas"[All Fields])) AND ("neck"[MeSH Terms] OR "neck"[All Fields] OR ("cervic"[All Fields] OR "cervicals"[All Fields] OR "cervices"[All Fields] OR "neck"[MeSH Terms] OR "neck"[All Fields] OR "cervical"[All Fields] OR "uterine cervicitis"[MeSH Terms] OR ("uterine"[All Fields] AND "cervicitis"[All Fields]) OR "uterine cervicitis"[All Fields] OR "cervicitis"[All Fields]) OR ("head"[MeSH Terms] OR "head"[All Fields])) AND ("child"[MeSH Terms] OR "child"[All Fields] OR "children"[All Fields] OR "child s"[All Fields] OR "children s"[All Fields] OR "childrens"[All Fields] OR "childs"[All Fields] OR ("paediatrics"[All Fields] OR "paediatrics"[MeSH Terms] OR "pediatrics"[All Fields] OR "pediatric"[All Fields] OR "paediatric"[All Fields]) OR ("infant"[MeSH Terms] OR "infant"[All Fields] OR "infants"[All Fields] OR "infant s"[All Fields]) OR ("infant, newborn"[MeSH Terms] OR ("infant"[All Fields] AND "newborn"[All Fields]) OR "newborn infant"[All Fields] OR "newborn"[All Fields] OR "newborns"[All Fields] OR "newborn s"[All Fields]) OR ("adolescences"[All Fields] OR "adolescency"[All Fields] OR "adolescent"[MeSH Terms] OR "adolescent"[All Fields] OR "adolescence"[All Fields] OR "adolescents"[All Fields] OR "adolescent s"[All Fields]))	45
Embase	March, the 3rd, 2022	('sirolimus'/exp OR sirolimus OR 'rapamycin'/exp OR rapamycin) AND ('lymphatic'/exp OR lymphatic OR vascular OR 'lymphangioma'/exp OR lymphangioma) AND ('neck'/exp OR neck OR cervical OR 'head'/exp OR head) AND ('child'/exp OR child OR 'paediatric'/exp OR paediatric OR 'infant'/exp OR infant OR 'newborn'/exp OR newborn OR 'adolescent'/exp OR adolescent)	104
Cochrane library	March, the 3rd, 2022	(sirolimus OR rapamycin) AND (lymphatic OR vascular OR lymphangioma) AND (neck OR cervical OR head) AND (child OR paediatric OR infant OR newborn OR adolescent) in Title Abstract Keyword—(Word variations have been searched)	1
Web Of Science	March, the 3rd, 2022	(sirolimus OR rapamycin) AND (lymphatic OR vascular OR lymphangioma) AND (neck OR cervical OR head) AND (child OR paediatric OR infant OR newborn OR adolescent) (all fields)	48
Clinicaltrials.gov	March, the 3rd, 2022	((sirolimus OR rapamycin) AND ( lymphatic OR vascular OR lymphangioma) AND ( neck OR cervical OR head) AND ( child OR paediatric OR infant OR newborn OR adolescent))	8
Scopus	March, the 3rd, 2022	TITLE-ABS-KEY ( ( sirolimus OR rapamycin) AND ( lymphatic OR vascular OR lymphangioma) AND ( neck OR cervical OR head) AND ( child OR paediatric OR infant OR newborn OR adolescent))	59
Total non-unique hits			265

We included studies in which sirolimus was used to treat LM that involved at a minimum the head and neck region of paediatric patients with reported treatment outcomes. We excluded meta-analyses and systematic and narrative reviews, which were nevertheless hand-checked for additional potentially relevant studies. No minimum study population was required. Mixed venolymphatic malformations were excluded from the review.

Abstracts and full texts by different authors were reviewed in duplicate. At the abstract review stage, we included all studies that were deemed eligible by at least one rater. At the full-text review stage, disagreements were resolved by achieving consensus among raters.

### PICOS criteria

The Population, Intervention, Comparison, Outcomes, and Study (PICOS) framework for the review was defined as follows:

- P: any paediatric patient with a simple lymphatic malformation involving the head and neck region.
- I: treatment with sirolimus, regardless of the administration route, dosage, and combination with other treatment(s).
- C: no comparator available.
- O: effectiveness of sirolimus treatment and adverse effects.
- S: all original study types, including case reports.

### Study assessment and data extraction

For each study included, we recorded the following: study type, number of sirolimus-treated LMs, female to male ratio, patients’ age, type of LM (i.e. micro-, macrocystic, or mixed), clinical response rate, LM volume reduction (rate and assessment type), LM size or De Serres stage [18], LM site, sirolimus administration route and dose, sirolimus-related adverse events, follow-up time, and other prior, concurrent, or further treatments (the latest along with their timing). Two authors extracted data and rated studies in duplicate, and disagreements were resolved by consensus. A clinical response was defined either as a significant LM reduction after sirolimus treatment and/or as an improvement in signs and symptoms caused by the LM after sirolimus treatment.

Studies were assessed for both quality and methodological bias according to the Joanna Briggs Institute Critical Appraisal tools (JBI-CAT) (for case reports) [19], the National Heart, Lung, and Blood Institute Study Quality Assessment Tools (NHI-SQAT) [20] (for case series and cohort studies), and the revised Cochrane risk-of-bias tool (for randomised clinical trials) [21]. Items were rated as ‘good’ if they fulfilled at least 80% of the items reported in the JBI-CAT or NHI-SQAT, ‘fair’ if they fulfilled between 50 and 80% of the items, and ‘poor’ if they fulfilled less than 50% of the items, respectively.

The level of evidence for clinical studies was scored according to the Oxford Centre for Evidence-based Medicine (OCEBM) level of evidence guide [9, 22].

Due to the considerable heterogeneity of study populations, study methods, and the predominantly qualitative nature of collected data, no initial meta-analysis was planned or performed a posteriori.

## Results

**Fig. 1:**
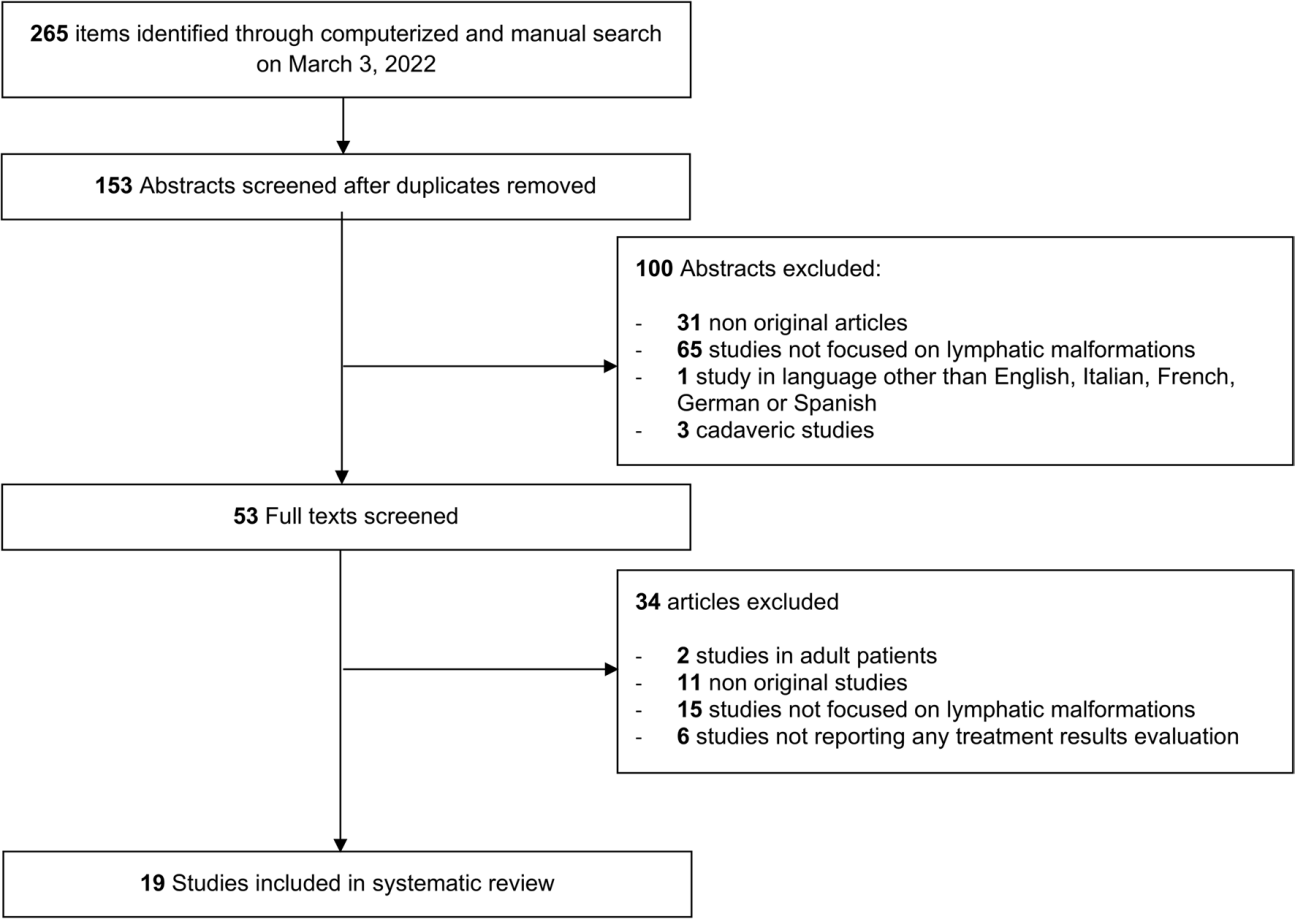
PRISMA-style flow diagram of study selection during the systematic review process

Among the 153 unique research items initially identified, 133 published reports were selected for full-text evaluation. No further report was identified for full-text evaluation after reference checking. Overall, 19 studies published between 2015 and 2021 were retained for analysis (see Fig. 1) [15, 23–40].

Nine articles were case reports, seven were case series, and three were prospective cohort studies. Their level of evidence according to the OCEBM scale was IV (*n* = 16) and II (*n* = 3). Clinical articles were rated as good (*n* = 7) or fair (*n* = 12) according to NHI-SQAT tools, with no article receiving a rating of low quality. No significant biases towards the objectives of our systematic review were identified. Table 2 shows the study type, evidence, and quality rating for all studies included. Reported evidence was available for sirolimus treatment in 97 patients with LM and without a clear gender prevalence (34 females, 32 males, and 31 patients with gender not reported). The age range was broad, from intrauterine to 192 months. There was no evident prevalence for macro- or microcystic LM, and the size or stage was unevenly reported. More, specifically, LM type was reported in 66 patients, among whom 24 had macrocystic lesions, 17 microcystic lesions and 25 mixed lesions. Most patients had LM extending from the neck to at least one other region, with the mediastinum and tongue being the most frequent. Clinical response to sirolimus treatment was reported in 89 cases, although volume reductions were reported inconsistently. The clinical response rate was 23 out 24 cases for macrocystic lesions, 14 out 17 for microcystic lesions, and 21 out of 25 for mixed lesions. The route of administration was oral in all reported cases. The most frequent dose was 0.8 mg/m^2^, with the aim of achieving a therapeutic blood concentration of 10–15 ng/mL. Upper respiratory tract infections and mouth sores were the most frequently reported sirolimus-related adverse events. Sclerotherapy was the most frequent treatment preceding, following, or coinciding with sirolimus. Surgery was the second most employed associated treatment modality. Follow-up was extremely variable, ranging from 6 months to 5 years. Table 3 shows the demographic and clinical information for the treated patients, and Table 4 shows the data on treatment regimens, adverse events, concurrent treatments, and follow-up.

**Table 2:** Type of study, and evidence and quality rating of reviewed articles

References	Study type	OCEBM rating	Quality rating
Alemi et al. [23]	CR	4	F
Cheng and Yoo [25]	CR	4	F
Cheng et al. [24]	CR	4	G
Francis et al. [26]	CR	4	F
Gaffuri et al. [27]	CS	4	F
Gómez Sánchez et al. [28]	CS	4	F
González-Hermosa et al. [29]	CR	4	F
Hammer et al. [30]	PCS	2	G
Holm et al. [31]	CS	4	F
Laforgia et al. [32]	CR	4	F
Livingston et al. [33]	CR	4	F
Meurisse et al. [34]	CR	4	F
Ozeki et al. [35]	PCS	2	G
Reinglas et al. [15]	CR	4	G
Strychowsky et al. [36]	CS	4	G
Triana et al. [37]	CS	4	G
Wu et al. [38]	CS	4	F
Zhang et al. [39]	PCS	2	G
Zobel et al. [40]	CS	4	F

*CR* case report, *CS* case series, *PCS* prospective cohort study, *OCEBM* Oxford centre for evidence-based medicine, *F* fair, *G* good

**Table 3:** Demographic and clinical information on the treated patients for all included studies

References	Treated patients (*n*)	Female:male ratio (*n*:*n*)	Patients’ age (mo)	Cystic type	LM size or De Serres stage	LM site	Clinically responding cases [*n* (%)]	Tumour volume reduction % (evaluation method)
Alemi et al. [23]	2	NR	1;1	Mi (*n* = 1); Ma (*n* = 1)	DSS V (*n* = 2)	Face, neck and mediastinum (*n* = 2), anterior thoracic wall (*n* = 1)	2 (100%)	NR
Cheng and Yoo [25]	1	1:0	23	Ma	NR	Neck	1 (100%)	Near complete resolution (MRI)
Cheng et al. [24]	1	0:1	1	NR	10 × 8 × 6 cm (thoracic portion)	Neck and chest	1 (100%)	NR
Francis et al. [26]	1	NR	0.6	Ma	NR	Neck	1 (100%)	NR
Gaffuri et al. [27]	1	1:0	NR	Ma	300 mL	Neck, mediastinum	0 (0%)	NR
Gómez Sánchez et al. [28]	3	0:3	108; 36; 96	NR	NR	Parotid region/ear (*n* = 1), neck (*n* = 2)	3 (100%)	NR
González-Hermosa et al. [29]	1	0:1	0.6	Mixed	NR	Floor of the mouth, both parotid regions and oropharynx, retropharyngeal region and neck	1 (100%)	NR
Hammer et al. [30]	3	2:1	36; 132; 192	NR	NR	Neck and larynx; neck and parotid region; retro-orbital region	3 (100%)	2%; NR; 6.7% (MRI)
Holm et al. [31]	12	5:7	Me 36.5; Ra 1–143	NR	NR	Extensive cervicofacial involvement (*n* = 11), tongue (*n* = 1)	10 (83.3%)	NR
Laforgia et al. [32]	1	1:0	0.3	NR	NR	Neck, mediastinum, liver (third segment), and bones	1 (100%)	100% (MRI)
Livingston et al. [33]	1	NR	30 weeks (foetal)	Ma	6 cm3	Face, neck and mediastinum	1 (100%)	NR
Meurisse et al. [34]	2	1:1	0.75; 2.25	Ma	NR	Neck (*n* = 2)	2 (100%)	70%;80% (clinical estimate)
Ozeki et al. [35]	5	3:2	0.5; 10; 12; 36; 132	NR	NR	Face (*n* = 1), face and neck (*n* = 1), neck and mediastinum (*n* = 2), neck, chest and abdominal cavity (*n* = 1)	4 (80%)	27.9%; 23.4%; 28.1; 24.3; 15% (MRI)
Reinglas et al. [15]	1	0:1	4	NR	NR	Neck and mediastinum	1 (100%)	NR
Strychowsky et al. [36]	16	NR	Me 70.5	Mi (*n* = 7), Mixed (*n* = 9)	NR	Face (*n* = 1), face and neck (*n* = 15)	16 (100%)	Av 26% (estimate on clinical photographs or NR radiological evaluation)
Triana et al. [37]	6	NR	Me 0.5	Ma	7 × 5,8 × 4,7 cm	Neck, mouth floor, and airway (*n* = 6), tongue (*n* = 4), mediastinum (*n* = 2)	6 (100%)	NR
Wu et al. [38]	8	5:3	Me 11.89 ± 13.11	Mi (*n* = 2), mixed (*n* = 6)	NR	face and neck (*n* = 5), face, neck, and mediastinum (*n* = 2)	8 (100%)	Av 29% (MRI)
Zhang et al. [39]	27	15:12	Av 27.37	Ma (*n* = 11), mi (*n* = 7), mixed (*n* = 9)	DSS II 25,9%, III 11,11%, IV 14,8%, V 7,4%	Face neck region or cervical–thoracic region	23 (85%)	Av 47.7% (MRI)
Zobel et al. [40]	5	NR	NR	NR	NR	face and neck	4 (80%)	NR

*NR* not reported (at least for the lymphatic malformation population), *Me* median, *Ra* range, *Av* average, *Mi* microcystic, *Ma* macrocystic, *DSS* De Serres Stage, *MRI* magnetic resonance imaging

**Table 4:** Treatment regimens, adverse events, and concurrent treatments for all included studies

References	Sirolimus administration route	Sirolimus dose	Sirolimus-related adverse events	Other prior treatments	Other concurrent treatments	Other further treatments (with timing if available)	Follow-up duration
Alemi et al. [23]	NR	NR	None	Sclerotherapy (*n* = 2), (sildenafil and propranolol, *n* = 1)	None	CO2 laser ablation (*N* = 1, 21 mo post Sirolimus)	10 mo; 3 y 1 mo;
Cheng and Yoo [25]	Oral	0.8 mg/m^2^ bid, TBC 10–15 ng/mL	NR	Sclerotherapy	Sclerotherapy	None	12 mo
Cheng et al. [24]	NR	NR	None	Sclerotherapy	Thoracoscopic resection of thoracic portion	None	12 mo
Francis et al. [26]	NR	NR	NR	None	Sclerotherapy	Sclerotherapy (14 days post sirolimus), surgery (before hospital discharge)	NR
Gaffuri et al. [27]	NR	0.8 mg/m^2^ bid, TBC 10–15 ng/mL	Transient recurrent nerve palsy	None	None	Surgery (1 mo post sirolimus)	NR
Gómez Sánchez et al. [28]	Oral	0.8 mg/m^2^ bid, TBC 10–15 ng/mL	Mild hypercholesterolemia, oral rash	Sclerotherapy (*n* = 3), surgery (*n* = 1), CO2 laser (*n* = 2)	None	None	4 y 4 mo; 8 mo; 19 mo
González-Hermosa et al. [29]	Oral	0.8 mg/m^2^ qd	None	None	None	Surgery (16 mo post sirolimus)	16 mo
Hammer et al. [30]	NR	0.8 mg/m^2^ bid, TBC 10–15 ng/mL	Mucositis	Surgery (*n* = 3), sclerotherapy (*n* = 1)	None	Sclerotherapy (*n* = 1, 13 mo post sirolimus)	34 mo, 13 mo, 28 mo
Holm et al. [31]	NR	0.8 mg/m^2^ bid, TBC 10–15 ng/mL	Ulceration at pigtail catheter site (*n* = 1), mild infections (*n* = 7), lymphopenia (*n* = 2), hypophosphatemia (*n* = 1)	Sclerotherapy (*n* = 10), surgery (*n* = 7), CO_2_ laser ablation (*n* = 8), radiofrequency ablation (*n* = 1)	None	bevacizumab (*n* = 1, 8 mo post sirolimus)	Range 8 mo – 5.6 y
Laforgia et al. [32]	Oral	0.8 mg/m^2^ bid, TBC 10–15 ng/mL	None	Propranolol	None	None	15 mo
Livingston et al. [33]	Oral (maternal until delivery)	Maternal: oral 15 mg load, then 5 mg qd TBC 5–15 ng/mL. Paediatric: not reported dose to achieve 5–15 ng/mL blood range	None	None	None	Sclerotherapy (3 weeks post sirolimus)	12 mo
Meurisse et al. [34]	NR	0.08 mg/kg qd, TBC 3.5–6 ng/mL (1 case), 4–12 ng/mL (1 case)	None	Macrocystic lesion aspiration (*n* = 1), sclerotherapy (*n* = 2), sildenafil (*n* = 1)	None	None	22 mo, 3 y
Ozeki et al. [35]	Oral	2 mg qd in patients with BSA ≥ 1.0 m^2^, 1 mg qd in patients with BSA ≥ 1.0 m^2^, target therapeutic range 5–15 ng/mL	Cellulitis (*n* = 1), stomatitis (*n* = 1), URTI (*n* = 1)	Sclerotherapy and Chinese medicinal herbs (*n* = 3), blood transfusion (*n* = 1), steroids and propranolol (*n* = 1)	None	None	14 mo; 6 mo; 18 mo; 6 mo; 6 mo
Reinglas et al. [15]	Oral	0.4 mg/m^2^ bid, TBC 5–10 μg/L	Mild hypertension	None	None	None	18 mo
Strychowsky et al. [36]	NR	0.8 mg/kg qd, TBC 10 and 15 ng/mL, further shifted to 7 to 13 ng/mL based on mild toxicities at higher levels	Cellulitis, eczema (*n* = 6), emesis/nausea (*n* = 3), neutropenia (*n* = 3), mouth sores (*n* = 9), diarrhoea (*n* = 1), elevated cholesterol and triglycerides (*n* = 3), transaminitis (*n* = 5), rash (*n* = 1), irregular menstrual bleeding (*n* = 2), joint pain (*n* = 1), fatigue (*n* = 1)	Sclerotherapy	None	Sclerotherapy, surgery (*n* and timing NR)	Range 10 mo–4 y
Triana et al. [37]	NR	0.8 mg/m^2^ bid, TBC 4–12 ng/mL	Hypertriglyceridemia (*n* = 1), elevation of gamma-glutamyl transferase (*n* = 2)	None	None	Surgery (*n* = 2, timing NR)	30 mo; 41 mo; 46 mo; 31 mo; 4 mo; 4 mo
Wu et al. [38]	Oral	0.8 mg/m^2^ bid, TBC 10–15 ng/mL	Mouth sores (*n* = 6), eczema (*n* = 1), gastrointestinal reaction (*n* = 2), dyslipidaemia (*n* = 1), upper respiratory infection (*n* = 1), neutropenia (*n* = 1)	Sclerotherapy and/or surgery	None	None	12 mo
Zhang et al. [39]	Oral	0.8 mg/m^2^ bid, TBC 4–13 ng/mL	Mucositis (*n* = 5), hypercholesterolemia (*n* = 5), upper respiratory infection (*n* = 7), hepatic dysfunction (*n* = 6), dizziness (*n* = 1), cystic haemorrhage (*n* = 2)	None	None	None	Range 6–27 mo
Zobel et al. [40]	NR	0.8 mg/m^2^ qd, titrated to goal trough level between 10 and 15 ng/mL	Nausea (*n* = 1)	NR	None	Sclerotherapy (*n* = 2, timing NR)	Mean 27.5 mo

*NR* not reported (at least for the lymphatic malformation population), *TBC* target blood concentration, *bid* bis in die, *qd* quaque die, *y* years, *mo* months

## Discussion

Our systematic review, the first to focus on the role of sirolimus treatment for paediatric head and neck LM, confirms a growing interest in this therapeutic approach and mirrors the encouraging results obtained with sirolimus alone or in combination with surgery and sclerotherapy.

Starting with, the pioneering work of Reinglas [15], case reports progressively gave way to more complex studies, culminating in a prospective study by Zhang et al., published in 2021 [39], thereby highlighting the interesting role for sirolimus in treating this challenging condition.

On one hand, the encouraging results provided by our systematic review (i.e. 89 out of 97 cases reported satisfactory treatment response) support the role of sirolimus in treating LM in children. Indeed, most studies included were of good or fair methodological quality. On the other hand, the lack of data from randomised controlled trials and the small size of the included case series underscores significant grey areas in the use of sirolimus for paediatric LM.

First and foremost, there is a general lack of awareness in reporting patient data. Published studies lacking basic demographic information—or not allowing for the extraction of the data for subpopulations such as gender and age—were far too common in our review. It is clear from a medical standpoint that an infant a few months old might differ from a teenager, not only in terms of disease history but also from a more general perspective, and this difference plays a substantial role in planning complex treatment strategies. Even worse, the cystic type of the malformation, as well as its size, stage, and symptoms were often inconsistently reported, making it difficult to obtain a better understanding of which patients are more suitable for this type of treatment. While Zhang et al. [39] reported a significantly better response for macrocystic LM, good clinical response rates were also reported across microcystic and mixed LM studies. These findings are consistent with our extrapolated data, which did not show an obvious higher clinical response rate in any single type of LM. Furthermore, this lack of definition makes the assessment of the treatment results more intricate, and much is left to the authors’ interpretation. Also detrimental to the understanding of the results was a lack of use of the Cologne Score [41], an assessment that was developed to quantify the functional burden of LM in terms of disfigurement, dysphagia, dysphonia, and dyspnoea.

This present systematic review also explores the role of sirolimus in broader treatment protocols for LM. Given the relatively recent introduction of sirolimus as a therapy for LM, it is not surprising that in about half the studies included, sirolimus was introduced only after treatment failure or symptom recurrence with other more frequently employed treatment options, such as sclerotherapy [42, 43]. Even more interesting, albeit only occasionally reported [25, 26], was the concurrent use of sirolimus and sclerotherapy without related adverse events. Given the ethical concerns in treating such a rare and serious condition in children, the sclerotherapy/sirolimus combination could represent a starting point for a well-designed randomised trial that would shed some light on the specific role of sirolimus in the course of LM. Further treatments following sirolimus therapy typically fall into two groups, ablation/demolition, for which sirolimus acts as a neoadjuvant therapy, or further sclerotherapy, with sirolimus acting to stabilise the LM growth or reduce the overall treatment volume.

The data emerging from the systematic analysis of sirolimus-related adverse events are far more complex. For a simple analysis, as shown in Table 4, it is apparent that the frequency of reported adverse events was proportional to the size of the patient population in each study, with case reports mentioning few to no complications, and larger case series such as that by Strychowsky et al. and Zhang et al. reporting 2.13 and 0.93 events, respectively, per treated paediatric patient [36, 39]. The reduced incidence of adverse events in case reports seemed to point towards a degree of reporting bias. Consequently, even if most events were mild, primarily with mucosa- or skin involvement, we advocate for proactive surveillance, particularly in the paediatric population. Such surveillance is even more critical, and inextricably connected to treatment efficacy evaluation, if we consider that wider reports suggest a lower incidence of adverse events with lower target plasma concentrations of sirolimus [44]. Preliminary data from our review show that such lower plasma concentrations also retain their effect for LM.

In the context of this systematic review, we strived to minimise bias in the selection of articles and extraction of the data. With this in mind, we did not choose time limits for our searches and included all article types to maximise the knowledge base. Nevertheless, we are indeed aware that the inclusion of case reports introduces a significant publication bias towards good results (or as a general rule, based on this review), extremely dire adverse events [45]. We believe that allowing for such bias was a fair compromise due to the lack of literature focusing on this rare disease. Another limitation of our study was the impossibility of assessing therapeutic success objectively, as neither volume reductions nor other specific metrics were consistently reported across articles. Instead, we chose to report the clinical response rate, adhering to each article authors’ view on what could be considered as such. This need for consistent reporting (which should start with using De Serres stages [18] and the Cologne Score System) is a major feature upon which future studies should focus. Last, but not least, to minimise heterogeneity, we excluded from this review venolymphatic malformations and complex LM, which are addressed even more sparsely in the literature but remain a potential target for sirolimus therapy. In addition, a few crucial aspects were missing from the data reviewed. First, the nature and degree of sirolimus-related infections, which are feared and frequently discussed complications. Even more importantly, there were no data on the long-term risks of treatment with sirolimus in the paediatric population. In fact, there no data were reported on the ideal duration of treatment, with some authors advocating for a ‘short course’ of 6–12 months and others suggesting the need for a treatment duration of indefinite length. This discrepancy raises potential concerns that are implicit based on the biological action of the drug.

## Conclusion

Our findings confirm that sirolimus is a potential treatment for simple LM, with encouraging clinical response rates and manageable side effects. Further characterisation of affected patients and LM, including an improved definition of therapeutic ranges and a systematic evaluation of treatment results would allow for a more targeted selection of candidates, with improved overall results. We advocate further for multicentre and ultimately randomised, studies on sirolimus treatment for LM, and for improved care and effective treatment options for this vulnerable paediatric population.

## Data Availability

All data pertaining to this systematic review are available from the corresponding author upon reasonable request.
